# A Simultaneous Measurement Sensor for Temperature and Curvature Based on Congruent Quasi-Helical Long-Period Fiber Grating

**DOI:** 10.3390/s24175621

**Published:** 2024-08-30

**Authors:** Haoen Sun, Zaiqiang Gong, Xiangjie Qin, Wenhao Shen, Haiqin Ma, Qiuhong Pan, Chengguo Tong, Cheng Yuan

**Affiliations:** 1 Research Center for Advanced Rechnology of Marine Information, Guilin University of Electronic Technology, Beihai 536000, China; 22172301005@mails.guet.edu.cn (H.S.); gzqgzq2742@163.com (Z.G.); 22022303110@mails.guet.edu.cn (X.Q.); zc1567838@163.com (W.S.); 2 School of Electronic Information, Guilin University of Electronic Technology, Beihai 536000, China; yejunming@guet.edu.cn (H.M.); panqiuhongyc@163.com (Q.P.); 3 Guangxi Key Laboratory of Wireless Wideband Communication and Signal Processing, Guilin University of Electronic Technology, Guilin 541004, China

**Keywords:** optical fiber sensor, long-period fiber grating, curvature, temperature, two parameters, simultaneous measurement

## Abstract

This article presents a long-period fiber-grating sensor based on a congruent quasi-helical structure (CQH-LPFG) with the two-parameter measurement of both temperature and curvature. The CQH-LPFG sensor was manufactured using a high-frequency CO_2_ laser, and an innovative quasi-helical structure was introduced into the two-parameter measurement of the temperature and curvature of the optical fiber sensor with excellent results. The experiment and analysis demonstrate that the curvature sensitivities of the three resonance peaks in the 1440 nm to 1540 nm transmission spectrum were 11.88 nm/m^−1^, 8.05 nm/m^−1^, and 11.11 nm/m^−1^, and the curvature varied ranging from 0.156 m^−1^ to 0.494 m^−1^. The three resonance peaks showed temperature responsivities of 29.87 pm/°C, 24.65 pm/°C, and 36.85 pm/°C, respectively, and the linear fit was of excellent quality. In the case of measuring both curvature and temperature changes simultaneously, the resonant peak wavelength of the CQH-LPFG sensor was demodulated through matrix analysis, with dip A and dip C providing superior simultaneous measurements. These features make it a promising candidate for applications such as engineering machinery and the health inspection of buildings.

## 1. Introduction

At present, fiber optic sensors possess several advantages, including small size, immunity to electromagnetic interference [1], adaptation to harsh environments [2], and other beneficial features, for the industrial measurement field. These sensors exhibit considerable potential for advancement in fields, such as aerospace [3], engineering machinery [4], and building structural health monitoring [5]. The grating period length of long-period fiber grating (LPFG) exceeding 100 μm [6] results in relatively low accuracy requirements for production equipment [7], which facilitates mass production. These characteristics, combined with a high degree of sensing accuracy, contribute to the outstanding advantages of LPFG sensors in engineering machinery and other manufacturing industries. The periodic modulation of the refractive index within the core or cladding of a fiber along its propagation direction is used in LPFG sensors, which leads to high sensitivity, high precision, and excellent wavelength-division multiplexing capability.

Therefore, numerous scholars have investigated various sensors based on LPFG sensing theory. These studies demonstrate that LPFG-based sensors effectively detect various physical quantities, including temperature, curvature, strain, pressure, humidity, magnetic field strength, refractive index, and torsion [8,9,10,11,12]. Micromachining equipment, such as excimer lasers, femtosecond lasers, CO_2_ lasers, and ion-implantation lasers [13,14,15], can be used to fabricate long-period fiber-grating sensors. Furthermore, as technology advances, structures, such as single title [16], phase shift [17], chirped [18], twist [19], and helical [20], can be carved into optical fibers, bringing new directions in sensor fabrication.

Compared to mechanical and electronic sensors in health inspections for machinery and construction buildings, fiber optical sensors show advantages, particularly in monitoring curvature and temperature changes within the structure, which are essential. Optical sensors have significant advantages, such as high accuracy, resistance to electromagnetic interference, environmental adaptability, high sensitivity, long-distance measurement, and multiparameter measurement, as well as miniaturization and integration, making them the first choice for many demanding applications. Researchers have developed various fiber optical sensing techniques to fabricate fiber sensors capable of the two-parameter measurement of curvature and temperature. In 2017, David Barrera et al. developed a seven-core optical-fiber long-period grating sensor with a linear response in the curvature size region of 0 m^−1^ to 1.77 m^−1^ [21] and a curvature sensitivity of −4.85 nm/m^−1^. A frequency-doubled argon–ion laser was used to inscribe different long-period gratings on different cores of the seven-core optical fiber. Yang Yu et al. fabricated a negative curvature hollow-core fiber (NCHCF) [22]-based curvature sensor with a graded index–fiber (GIF)–NCHCF cascade structure in the presence of an anti-resonance (AR) mechanism and multimode interference (MMI), which showed a curvature correlation of −5.27 nm/m^−1^ in the region of 6.068 to 12.815 m^−1^. Zhao Yunhe et al. designed sensors with long-period gratings (LPGs) induced in a linearly arranged three-core fiber (TCF) using the electric arc–discharge technique; the curvature sensing responses of TCF-LPG were 7.58 and −8.83 nm/m^−1^ at two resonance dips of 1294 nm and 1535 nm in the range of bending from 0.249 to 0.8731 m^−1^ [23], respectively. However, although all of the above sensors are capable of curvature measurement, they have less sensitivity, more complex production methods, and higher production costs, and cannot realize simultaneous measurements, which prevents them from being applied in large numbers to various industries. Utilizing a CO_2_ laser to manufacture optical fibers simplifies the process. The laser writing process for optical fibers does not rely on specialized devices and complex equipment, making large-scale production more convenient. CO_2_ laser treatment reduces additional damage to the grating region of the optical fiber, improving production consistency and product quality. Sensors based on the simultaneous inscribing of three gratings to form a congruent quasi-helical structure in a single-mode fiber were applied to [24] the two-parameter [25] measurement of curvature and temperature. Although LPFG sensors are excellent at measuring a single physical parameter, their multiple parameters have sensitivities that complicate multiparameter measurements. As resonant peaks can have variable responses to multiple parameters, special grating designs or additional methods of demodulating the signal are required to achieve effective multiparameter measurements.

A long-period fiber-grating sensor for simultaneous two-parameter measurement is presented in this paper, with the congruent quasi-helical structure fabricated with a CO_2_ laser and tested for curvature and temperature. During optical propagation within the LPFG, the different grating periods and fiber characteristics lead to different modes of the core and cladding coupled to each other, and the periodicity of the grating changes so that the refractive index also changes [26]. Under the above conditions, different wavelengths generate distinct resonance peaks, which can be utilized for sensing measurements. The CQH-LPFG structure was fabricated in this study by the CO_2_-exposure method to inscribe three sides of the single-mode fiber, and the angle size of each two grooves was 120°, resulting in the cross-section of three gratings arranged in a congruent triangular pattern, approximating a helical structure. Through theoretical analysis and experimental verification, the results show that the CQH-LPFG sensor responds sensitively to changes in curvature and temperature. Its linear correlation between curvature and temperature sensing is excellent and demonstrates outstanding transmission stability. Furthermore, matrix analysis was utilized to demodulate simultaneous temperature and curvature measurements, where the optimum temperature and curvature resolutions were 0.05 °C and 4.15 × 10^−5^ m^−1^, respectively.

## 2. Fabrication and Principle

**Figure 1:**
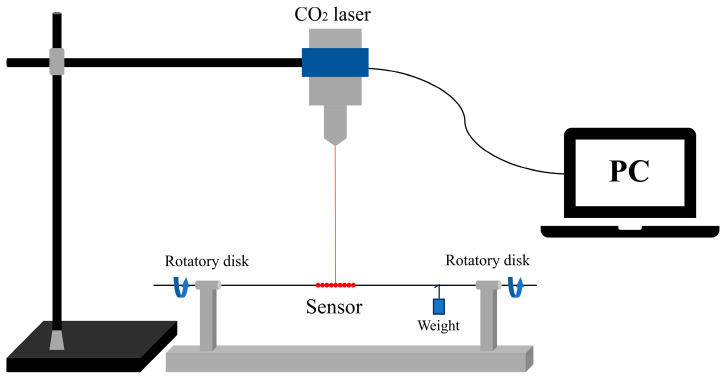
Schematic diagram of CQH-LPFG sensor fabrication device.

This novel congruent quasi-helical structure was fabricated to be manufactured by high-frequency CO_2_ lasers to produce a long-period fiber-grating sensor. The CQH-LPFG sensor was manufactured using single-mode optical fibers manufactured by Changfei Company. The fiber cladding and fiber core diameters were 125 µm and 9 µm, respectively. The schematic diagram of the experimental device for the fabrication of the CQH-LPFG sensor is shown in Figure 1. Three steps were necessary to fabricate this sensor. Initially, approximately 20 cm of a single-mode fiber was cut, and approximately 4 cm of the coating from the center of the single-mode fiber was removed using stripping clamps and cleaned with a cotton ball soaked in alcohol. Subsequently, the fiber was put into a fiber optic rotator, the rotator scale was adjusted to 0° after the fiber was clamped securely, and a 5 g weight was hung up to counteract the stresses applied during the engraving process. In the third step, the CQH-LPFG was carved with 45 grooves, symbolized by 1 to 45. Fifteen grooves (including 1, 4……40; 43) were carved into a single-mode fiber using a CO_2_ laser. The rotator was carved at intervals of a 120° rotation, and an additional 15 grooves (including 2, 5…41; 44) were carved at a position of 120° from the initial engraving. Similarly, when the two discs were rotated to 240°, the last set of gratings (including 3, 6…42; 45) was inscribed in the single-mode fiber. Whenever the optical fiber was rotated by a certain angle, we used the control system of the high-frequency CO_2_ laser to make the grating program move the same distance in the same direction, so that the grating could be inscribed again on the optical fiber fixed on the rotator. By combining the above process of rotating and writing the grating, we successfully fabricated a sensor (CQH-LPFG) with a congruent quasi-helical structure. With this approach, it was considered that three CO_2_ lasers could inscribe the fiber at the same time, and this LPFG was fabricated using this three-sided exposure method.

**Figure 2:**
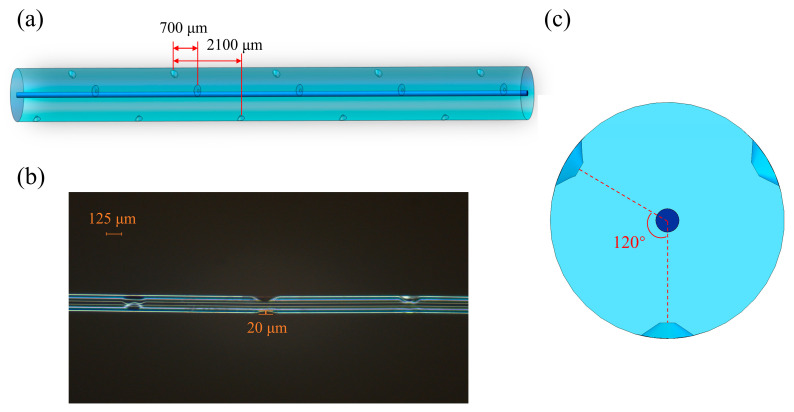
Schematic diagram of the CQH-LPFG sensor. (**a**) Schematic of sensor structure. (**b**) Cross-section of the CQH-LPFG sensor. (**c**) The CQH-LPFG sensor single-cycle micrograph of observation.

**Figure 3:**
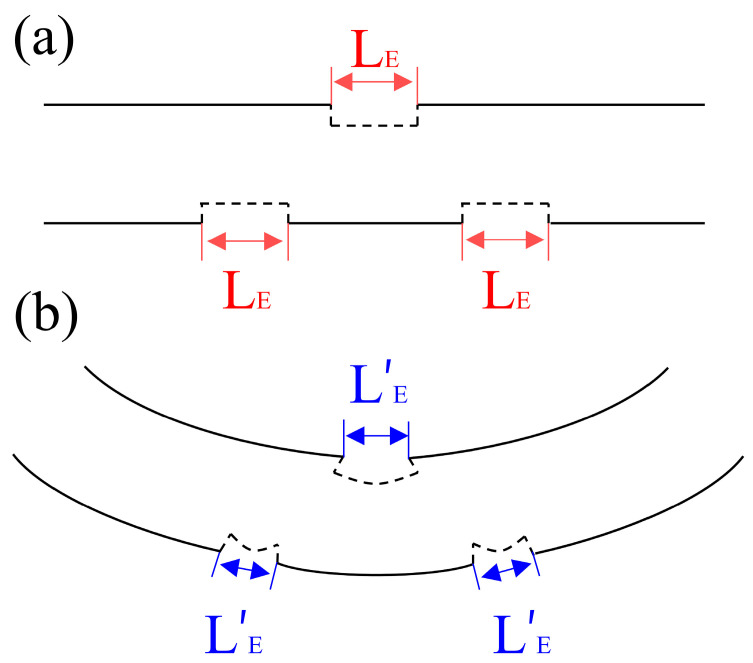
Bending grating deformation of the CQH-LPFG structure. (**a**) Schematic diagram of the horizontal state of a single quasi-helical grating structure. (**b**) Schematic diagram of the bending deformation of a single quasi-helical grating structure.

The distance between two axially neighboring gratings of the CQH-LPFG sensor was about 700 μm, and the length of one quasi-helical cycle was 2100 μm, as depicted in Figure 2a. In the cross-section, a helical structure with approximately congruent triangles was observed, and the angle between the two neighboring gratings was 120°, as depicted in Figure 2b. The groove depth etched by the CO_2_ laser was 15 μm, as shown in Figure 2c. The CQH-LPFG length was almost 31.5 mm. Since this CQH-LPFG sensor has a novel congruent quasi-helical structure, the cladding exhibited an asymmetric structure during the helical cycle, and each grating was deformed to different degrees during bending/straightening deformation [27], as illustrated in Figure 3. The fiber was subjected to local deformation during the grating inscription of the fiber by the CO_2_ laser [28]. The CQH-LPFG sensor was observed locally modulating the cladding of the fiber, which changed the refractive index of the cladding [29] and contributed to variations in the single-mode fiber core and cladding effective refractive indices at this location, significantly altering the fundamental model of the coupling between the core and the cladding.

According to the phase shift theory, the refractive index change resulting from sensor modulation causes a phase shift [30], which can be calculated using the following formula:

(1) $$\Phi =\frac{2\pi }{\lambda }\Delta n_{eff}^{cl,g}L_{E}$$

where the center wavelength is denoted as *λ*, the effective refractive index of the fiber cladding changes after inscribing the grating is Δ$n_{eff}^{cl,g}$, and the length of the inscribed grating notch is *L_E_*, as shown in Figure 3a. The bending-deformed grating notch length is *L’_E_* in Figure 3b. Figure 3a and Figure 3b represent the variations of the quasi helical structures in the horizontal and bent gratings, respectively. The phase matching condition of the CQH-LPFG is similar to that of a general LPFG:

(2) $$\lambda_{res}=(n_{eff}^{co}-n_{eff}^{cl,m})\Lambda$$

where $n_{eff}^{co}$ and $n_{eff}^{cl,m}$ indicate the effective refractive indices of the optical fiber core fundamental mode and cladding M-order modes, respectively, *λ_res_* denotes the resonant wavelength, and *Ʌ* is the grating period. When the outside environment of the sensor was changed, the wavelength change in the harmonic peak was primarily associated with the change in the effective refractive index of the CQH-LPFG. The difference in the effective refractive indices in the core fundamental mode and the cladding mode in the fiber was changed accordingly. Differing effective refractive indices can be expressed as:

(3) $$\Delta n=\Delta n_{eff}+(\Delta n_{eff}^{co}-\Delta n_{eff}^{cl,m})$$

where Δ*n* and Δ*n_eff_* are the difference between the effective refractive indices of the core and cladding and the difference between the effective refractive indices of the core base mode and cladding mode, respectively. Δ$n_{eff}^{co}$ and Δ$n_{eff}^{cl.m}$ are the differences between the effective refractive indices of the core and cladding, respectively, caused by the change in the external environment. The optical transmission mode and normalized frequency of the fiber optical sensor are the primary factors affecting the amplitude and direction of the resonance peak drift. Fiber optical materials are subject to thermo-optic and thermal expansion effects during temperature variations. These effects cause a change in the refractive index of the core and cladding, resulting in a displacement in the resonance peak, as shown in the following:

(4) Δλ=λ(α+ξ)ΔT

where Δ*λ* represents the amount of change in the resonant wavelength, *λ* is the initial resonant wavelength, *α* shows the thermo-optic coefficient of the fiber, *ξ* is the thermal expansion coefficient of the fiber, and Δ*T* is the amount of change in the temperature. The free spectrum (FSR) range of the CQH-LPFG was also derived by using Taylor Expansion Theory with the following equation:

(5) $$FSR=\lambda^{2}/(n_{eff}^{co}-n_{eff}^{cl,m})H$$

where *H* denotes the effective interference length. Based on the above analysis and the coupled-mode theory, the effective interference length approximately equals the total length of the fiber grating, which is 31.5 mm. The difference in the effective refractive indices of the gratings was 0.0507, 0.0543, and 0.0561, respectively, and the grating period length was 2.1 mm. The refractive indices of the core and cladding of the single-mode fiber were 1.467 and 1.456, respectively. In the ideal state of light propagation through the optical fiber, the modes are orthogonal to each other and are not coupled together. However, fiber grating alters its optical properties, resulting in mode coupling, allowing the CQH-LPFG sensor to form three resonant peaks that can be used for sensing measurements. Therefore, through the above theoretical analysis, with changes in physical quantities sensed in the sensor,, the CQH-LPFG change in physical quantities around the grating led to the resulting change in the effective refractive index of the core and cladding, allowing the transmission spectrum of the CQH-LPFG to be altered. As the curvature increased, this CQH-LPFG experienced a redshift phenomenon at the resonance peak. A similar increase in temperature also resulted in a redshift at the resonance peak.

## 3. Experimental Results

### 3.1. Experimental Setup

Based on the sensor fabrication process, the temperature and curvature variations of this long-period fiber-grating sensor with a congruent quasi-helical structure were designed and analyzed. A super-continuous light source (CS-5) with a wavelength range from 450 to 2400 nm and a spectral output power float range of ±0.1 dB was used in the experiment. A Yokogawa model AQ6370D spectrometer was employed for the analysis, exhibiting a wavelength display range of 600–1700 nm and supporting a wavelength resolution of up to 0.02 nm.

### 3.2. Curvature Experiment

**Figure 4:**
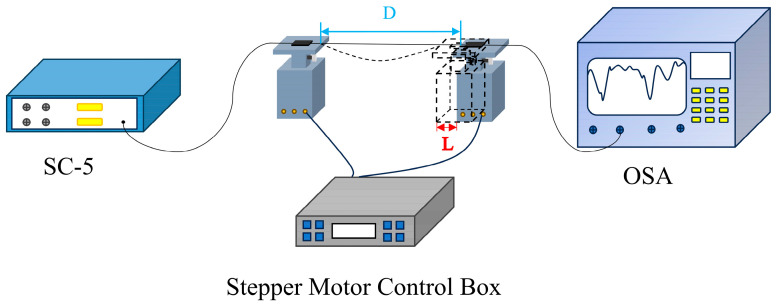
Schematic diagram of the curvature experiment devices.

As shown in Figure 4, the congruent quasi-helical long-period fiber-grating sensor was connected to the curvature experimental setup so that the sensor was located in the center of the two precision displacement stages. A broad-spectrum light source (CS-5) was connected at the left end, while an optical spectrum analyzer (OSA) was connected at the right to capture interference spectra. Precision displacement stages (accuracy: 1 μm) were used to change the curvature at the position of the CQH-LPFG sensor; one side of the displacement stage remained stationary, while the other side of the displacement stage was controlled by a controller equipped with stepper motors affecting the change. The magnitude of curvature corresponding to the sensor at various displacements was calculated using the following equation:

(6) $$C=\frac{1}{R}\approx \sqrt{\frac{24L}{D^{3}}}$$

where *C* is the curvature value, *R* is defined as the radius of curvature, *D* is the length between the two fiber grippers when the optical fiber is horizontal, and *L* is the displacement of the precision displacement stage. In the experiment, we used stepper motors to control the displacement stage to change the curvature of the position of the sensor by regularly changing the step size *D*, and, after each change, the optical spectrum analyzer was used to observe the variations in the resonant peaks of the transducer.

**Figure 5:**
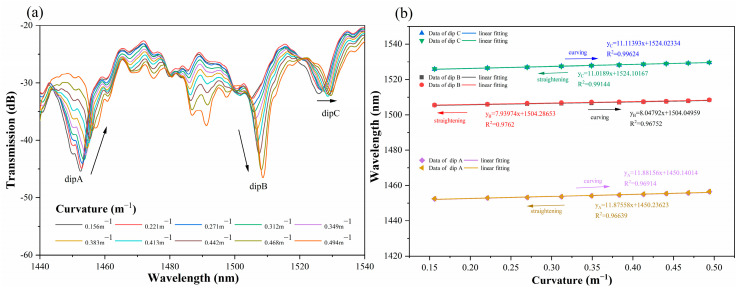
(**a**) Transmission spectra of CQH-LPFG for different curvatures. (**b**) Linear fitting results for dip A, dip B, and dip C bending and straightening.

The experimental results demonstrate that, from 0.156 to 0.494 m^−1^, the resonance peaks of dip A, dip B, and dip C of the congruent quasi-helical sensor on the spectrometer realistically drifted toward the long-wave direction (redshift phenomenon), as shown in Figure 5a, when the curvature changed. Interference depression and wavelength drift together affect the curvature sensitivity of LPFG sensors. At longer wavelengths, the interference depression is more susceptible to alterations in the refractive index of the fiber and grating period variations, resulting in heightened mode coupling effects and more significant interference phenomena.

The curvature responsiveness of dip A, dip B, and dip C was measured as 11.88 nm/m^−1^, 8.05 nm/m^−1^, and 11.11 nm/m^−1^, as shown in Figure 5b. Throughout the bending and straightening process of the CQH-LPFG sensor, the transmission spectrum reflects minimal variations in the curvature change sensitivity, with a relatively low hysteresis response in the experimental tests. These characteristics contribute to the sensor’s promising potential for curvature measurement and test repeatability.

### 3.3. Temperature Experiment

**Figure 6:**
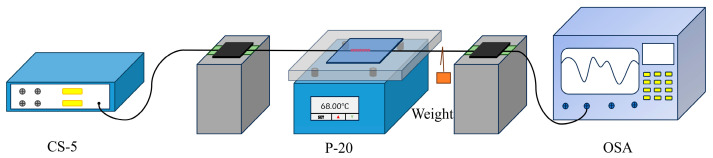
Schematic diagram of the temperature experiment devices.

The schematic diagram of the temperature experiment is shown in Figure 6. The left of the CQH-LPFG sensor was linked to the supercontinuum light source (SC-5), and the spectrum analyzer was connected to the right side so that all the sensors were placed in the center of the thermostatic platform (P-20). The CQH-LPFG sensor was shielded with an insulation board to avoid interference with the variation in external physical quantities in the experiments. On the right side of the optical fiber, a weight was hung so that the optical fiber maintained a straight state to avoid the influence of external environmental factors at the same time and prevent the fiber from curving due to the rising temperature, which could interfere with the experiments and lead to deviations in the experimental results. The experimental test from 28 °C to 98 °C and the wavelength deviation of the transmission spectra were documented for every 10 °C of temperature variation.

**Figure 7:**
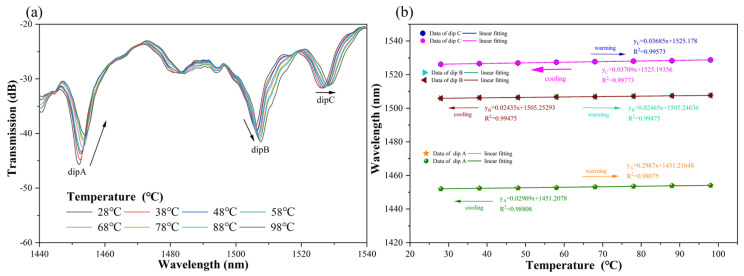
(**a**) Transmission spectra of CQH-LPFG for different temperatures. (**b**) Linear fitting results for dip A, dip B, and dip C warming and cooling.

The analysis of the experimental results reveals that the three resonance peaks dip A, dip B, and dip C, illustrated in Figure 7a, drifted toward the direction of longer wavelength (redshifted), which indicates it was a result of the increasing environmental temperature in which the CQH-sensor was positioned. The three resonant peaks, dip A, dip B, and dip C, were fitted, as shown in Figure 7b. The temperature responses of the resonant peaks of dip A, dip B, and dip C were 29.87 pm/°C, 24.65 pm/°C, and 36.85 pm/°C, respectively, and their linear correlations were excellent. The temperature sensitivities of their transmission spectral responses were also nearly the same in the warming and cooling experiments, and there was no hysteresis. This indicates excellent temperature measurement accuracy and repeatability.

### 3.4. Simultaneous Measurement

The analysis of the experimental results indicates that the CQH-LPFG sensor has excellent curvature and temperature responses to curvature and temperature at the locations where dip A, dip B, and dip C were located. The resonant peaks dip A, dip B and dip C simultaneously changed at different curvatures and temperatures, and the amount of resonant peak drift can be expressed by the following equation:

(7) $$\Delta \lambda_{A}=K_{Cr-A}\Delta C+K_{Te-A}\Delta T$$

(8) $$\Delta \lambda_{B}=K_{Cr-B}\Delta C+K_{Te-B}\Delta T$$

(9) $$\Delta \lambda_{C}=K_{Cr-C}\Delta C+K_{Te-C}\Delta T$$

where Δ*λ_A_*, Δ*λ_B_*, and Δ*λ_C_* represent the wavelength drifts of dip A, dip B, and dip C, respectively, and Δ*T* and Δ*C* measure the changes in temperature and curvature. *K_Cr-A_*, *K_Cr-B_*, and *K_Cr-C_* represent the curvature response factors of peaks A, B, and C of the CQH-LPFG, and *K_Te-A_*, *K_Te-B_*, and *K_Te-C_* represent the temperature response factors of peaks A, B, and C of the CQH-LPFG. From the above equation, we calculated the temperature and curvature change in the curvature by the magnitude of the wavelength displacement. Matrix analysis is a method for measuring and analyzing several environmental parameters simultaneously. We can understand its sensitivity by the above analysis. Therefore, when curvature and temperature change simultaneously, we could perform a two-parameter matrix analysis on the three resonance peaks simultaneously and, respectively, because of the dual sensing characteristics of the sensor for temperature and curvature. Adjusting the drift of the peaks by this demodulation method allows the temperature and curvature of the sensor to be accurately and simultaneously determined. The inverse matrix of the sensitivity response for each of the three peaks is shown below.

(10) $$\left[\begin{array}{l} \Delta C\\ \Delta T \end{array}\right]={\left[\begin{array}{l} K_{Cu-A}K_{Te-A}\\ K_{Cu-B}K_{Te-B} \end{array}\right]}^{-1}\left[\begin{array}{l} \Delta \lambda_{A}\\ \Delta \lambda_{B} \end{array}\right]=\frac{1}{52.39}\left[\begin{array}{l} 24.65\;-29.87\\ -8.05\;11.88 \end{array}\right]\left[\begin{array}{l} \Delta \lambda_{A}\\ \Delta \lambda_{B} \end{array}\right]$$

(11) $$\left[\begin{array}{l} \Delta C\\ \Delta T \end{array}\right]={\left[\begin{array}{l} K_{Cu-A}K_{Te-A}\\ K_{Cu-C}K_{Te-C} \end{array}\right]}^{-1}\left[\begin{array}{l} \Delta \lambda_{A}\\ \Delta \lambda_{C} \end{array}\right]=\frac{1}{105.92}\left[\begin{array}{l} \;\;36.85\;-29.87\\ -11.11\;11.88 \end{array}\right]\left[\begin{array}{l} \Delta \lambda_{A}\\ \Delta \lambda_{C} \end{array}\right]$$

(12) $$\left[\begin{array}{l} \Delta C\\ \Delta T \end{array}\right]={\left[\begin{array}{l} K_{Cu-B}K_{Te-B}\\ K_{Cu-C}K_{Te-C} \end{array}\right]}^{-1}\left[\begin{array}{l} \Delta \lambda_{B}\\ \Delta \lambda_{C} \end{array}\right]=\frac{1}{22.78}\left[\begin{array}{l} \;\;36.85\;-24.65\\ -11.11\;\;\;8.05 \end{array}\right]\left[\begin{array}{l} \Delta \lambda_{B}\\ \Delta \lambda_{C} \end{array}\right]$$

Based on the above matrix, it was calculated that the results of dip A and dip C are excellent for simultaneous measurements with a resolution of 4.15 × 10^−5^ m^−1^ and 0.05 °C for curvature and temperature, respectively. Additionally, we compared the CQH-LPFG sensor with other fiber optical sensors. As shown in Table 1, this sensor has a higher sensitivity and better temperature response in the micro-bending sensing field, and the equipment used is relatively simple, which is convenient for a significant level of promotion and utilization.

### 3.5. Sensor Measurement Error Analysis

**Figure 8:**
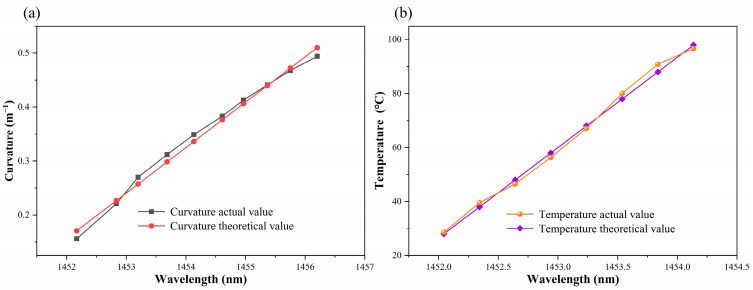
(**a**) Curvature corresponding to the actual and theoretical wavelengths of dip A. (**b**) Temperature corresponding to the actual and theoretical wavelengths of dip A.

**Figure 9:**
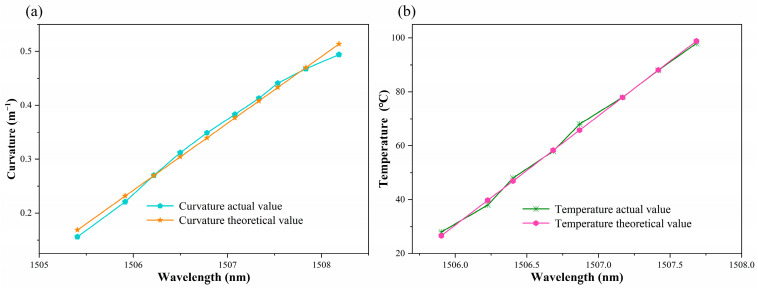
(**a**) Curvature corresponding to the actual and theoretical wavelengths of dip B. (**b**) Temperature corresponding to the actual and theoretical wavelengths of dip B.

**Figure 10:**
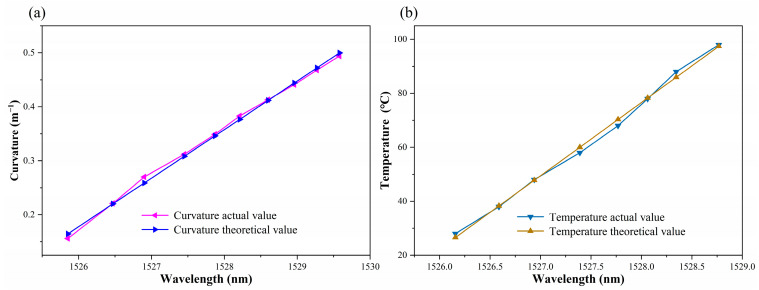
(**a**) Curvature corresponding to the actual and theoretical wavelengths of dip C. (**b**) Temperature corresponding to the actual and theoretical wavelengths of dip C.

The experimental analysis, as demonstrated in Figure 5b and Figure 7b, indicates that the sensitivity fitting equations for resonance peaks A, B, and C with respect to curvature and temperature could be obtained. Theoretical values of curvature and temperature corresponding to the actual resonance wavelength can be calculated utilizing the sensitivity fitting equations of sensor measurements. Furthermore, error analysis can be performed. Figure 8, Figure 9 and Figure 10 demonstrate the theoretical values of the resonant wavelength calculated from the fitted equations and their corresponding temperatures and curvatures, in addition to the actual measured wavelengths reflecting the temperatures and curvatures. The calculated results indicate that the curvature error range was less than 3%, and the temperature error range was less than 2.5%. In this study, the primary sources of experimental error were fluctuations in the light source output power, temperature fluctuations in the heating platform, and displacement errors in the curvature experimental device. The aforementioned combined errors resulted in discrepancies between the experimental results and the theoretical values.

## 4. Conclusions

In summary, this paper introduces a sensor for the two-parameter measurement of curvature and temperature based on long-period fiber-grating sensing technology fabricated with a CO_2_ laser in combination with a three-sided exposure method. Curvature sensitivities of 11.88 nm/m^−1^, 8.05 nm/m^−1^, and 11.11 nm/m^−1^ at dip A, dip B, and dip C ranged from 0.156 to 0.494 m^−1^. In contrast, the temperature sensitivities of 29.87 pm/°C, 24.65 pm/°C, and 36.85 pm/°C, respectively, were obtained in this experiment. Moreover, through matrix analysis, the sensor could simultaneously measure the two parameters of temperature and curvature. Dip A and dip C demonstrated superior simultaneous measurement capabilities, with a resolution of 4.15 × 10^−5^ m^−1^ and 0.05 °C for curvature and temperature, respectively. The CQH-LPFG sensor proposed in this paper features simple fabrication equipment with controllable and reproducible fabrication parameters and provides a novel preparation method to develop highly cost-effective LPFG sensors and is convenient for large-scale applications in the field of construction inspection and other fields.

## Figures and Tables

**Table 1:** Comparison of temperature and curvature sensitivity of different sensors.

Sensor Structure	Curvature Sensitivity	Range	Temperature Sensitivity	Ref.
MIs-CFIs	4.36 nm/m^−1^	0–1.134 m^−1^	71 pm/°C	[31]
ARROW-MZI	−4.28 dB/m^−1^	10.72–11.60 m^−1^	25.75 pm/°C	[32]
PSS-MZI	−0.68 nm/m^−1^	0.261–3.807 m^−1^	25.42 pm/°C	[33]
RCF-LPFG	0.879 dB/m^−1^	5–9.466 m^−1^	1.4 dB/°C	[34]
SCF-FBG	−3.91 nm/m^−1^	0.18–0.50 m^−1^	11.8 pm/°C	[35]
SCF-LPFG	−4.85 dB/m^−1^	0–1.77 m^−1^	/	[36]
THIS WORK	11.88 nm/m^−1^	0.156–0.494 m^−1^	36.85 pm/°C	/

## Data Availability

Data are contained within the article.
